# On the Mode of Action of Iodine and Its Preparations

**Published:** 1874-08

**Authors:** 


### ARTICLE V.

### *On the Mode of Action of Iodine and its Preparations.*

BY PROFESSOR SEE.

Iodine may be made to enter the system through different channels—viz., the digestive tube, the skin, the mucous membrane of the respiratory organs, and the serous cavities.

The digestive tube is the most certain and natural channel, and it is this which is nearly always taken advantage of. The tincture of iodine is scarcely ever prescribed internally—in fact, it possesses no advantages, but offers, on the contrary, certain inconveniences. If it remain in the stomach in the form of tincture, it produces a caustic effect on the mucous membrane of the digestive organ, but it always combines with a little soda or potash which it meets with in the stomach, and is converted into an iodated alkali. Hence it may be seen that those who administer iodine in its simple form are laboring under an erroneous impression if they imagine that the drug undergoes no change in the stomach.

The iodide of potassium should not be administered in the form of pills, as it is thus liable to produce a caustic effect on the mucous lining of the stomach; it should always be given in solution. And in prescribing this salt one should always bear in mind that the greater the quantity of liquid in which it is dissolved, the better the absorption. There is, however, a certain limit to the quantity of fluid to be employed, which of course a physician will not exceed, and which it is scarcely necessary even to mention.

The skin has often been selected as the channel for iodine to enter the economy. In employing an ointment composed of iodine in the proportion of one part to ten parts, in certain cases an effect is produced, in others nothing is obtained,—that is to say, in certain cases iodine has entered the organism, in others it has remained on the skin. It is expedient to know under what circumstances the iodine has been absorbed. Divers explanations have been given to account for the above facts. According to Professor See, two conditions contribute to the absorption of iodine:—1. To make iodine enter by the skin, the epidermis, which acts as a barrier, must be destroyed. To effect this, strong and repeated frictions of iodine ointment will have to be employed; but it is evident these cannot be continued, and a single friction would be perfectly useless. 2. In examining these facts, it is found that there are cases in which the epidermis has not been in the least affected by the frictions, and in which, nevertheless, the absorption of iodine might be proved. This would appear to be in contradiction to what has just been stated above, but it might be explained by the extreme volatility of this metalloid. When iodine is rubbed into the skin in the form of ointment, it is found in the mucous membrane of the lungs ; whereas when an ointment is made of an iodide, the latter is not found in the lungs, because it is not volatile, and does not contain free iodine Thus it may be seen it is by the air-passages, and not the skin, that the iodine entered the system ; and in proof that this is the case, it is sufficient to leave a phial of iodine uncorked near oneself, and the latter will be absorbed without touching or putting it to the nose, for it is found in the secretions.

Quacks seem to have been aware of this phenomenon when they invented the sachets of the powder of iodine, iodized cotton, and iodized flannel vests which are to be worn next the skin. These diverse agents possess a real therapeutic property; but the explanation of their action is the same as that given above— that is, the iodine they contain is absorbed by the air-passages, and not by the skin. If a piece of iodized cotton be placed on the arm, and covered with a watch glass or a glass bell, nothing will be observed ; but in a person who wears an iodized vest constantly, the iodine enters his economy, not by his skin, but by his nostrils.

Painting with the tincture of iodine has much the same action ; we know to what extent this is now employed, and there is scarcely a pain, a case of scrofula or phthisis, in which it is not resorted to. In phthisical patients, the tincture of iodine externally has taken the place of blisters and cauteries ; and the change is certainly to the advantage of the iodine ; but its action is not that of blisters or cauteries. Here, also, the same explanation may be given of its action ; but there is one effect which is scarcely suspected, and that is when the tincture is sufficiently strong, or the painting too frequently renewed, the epidermis is destroyed. The iodine enters the fissures thus formed, and produces inflammation of the cellular tissue, as has been observed at post-mortem examinations. To produce a more direct action on the tubercles of phthisical patients, it would certainly be preferable to place an open phial of the tincture of iodine on a table near the patient, as has been practiced by M. Piorry, in order that the iodine may be inhaled.

Iodine baths are also intended to act on the skin. These baths, which used to be much lauded, are now seldom or never employed, as their efficacy is very much questioned. It has been asserted that after an iodine bath this metalloid has been found in the urine. In this case, how did the iodine enter the body? Not by the skin, but by the air-passages; and even then such a result cannot be obtained unless the bath-room be hermetically closed, and the patient remain in the bath some time.

Fomentations are also intended as a means of effecting the absorption of certain medicaments into the tissues. These substances are varied, according to the effect desired —such as the tincture of iodine, laudanum, belladonna, &c. As with frictions, a real effect is sometimes obtained with fomentations, at other times none. This depends on the state of the skin, which is different in different individuals. If the skin be soft and pervious, iodine and the other substances may be absorbed, but it is difficult to know when the skin is in a favorable condition for absorption and when it is not. There exists normally on the skin an oily coating, which opposes the penetration of the iodide of potassium. A soap bath may remove this varnish, but it is immediately reproduced; and individuals who have greasy skins, whatever they may do, will never succeed in making their skin absorb the iodide.

The same may be said of baths composed of the monosulphuret of sodium. Little or nothing is absorbed unless the doors and windows are closed, for the sulphuretted hydrogen which is evolved is about the only active agent, as it is taken up by the respiratory apparatus. This would explain the superiority of the sulphurous waters—such as Luchon, Barges, which whiten on being drawn—over those that do not whiten, as Amelie-les-Bains. Iodized baths owe their efficacy to the iodine being absorbed by the respiratory organs.

There are some natural iodated waters, but they are rare in France; there are only those of Salins and Salies, in Bearn, and it must be admitted that they are not very rich in iodine. In Switzerland they have the waters of Saxony; in Prussia, those of Kreutznach. These latter cannot be replaced; they are those that contain the most iodide and bromide of potassium combined. Nevertheless, the French might still avoid going to Prussia by utilizing hot sea-water baths. The sea water, and particularly the sea air contain a certain proportion of iodine and bromine. But it must not be forgotten that this atmosphere does not extend very far, and that about 400 or 500 yards from the shore we get the breeze, but not the iodized air; to have the benefit of this, one must remain the whole day on the beach, or, what is still better, take up his residence on the sea coast.

When iodine enters the economy it is easily detected, and is almost immediately found in the urine and in the saliva; but the whole is not found at once. The elimination of iodine takes place more rapidly when it is administered in the form of iodide; but in whatever manner it is given, when the iodine enters the blood it combines with the potassium contained in the corpuscles; and as the salts of potash are very diffusable, it is not surprising to find iodine in the urine almost immediately it enters the blood. Iodine remains in the economy longer than one would be led to suppose, judging from its facile elimination, and it is found in the saliva after its presence has ceased to be detected in the urine. The elimination of iodine is intermittent, and it has been frequently seen that an individual who had been eliminating iodine that he had been taking, ceases to eliminate for some time and then begins again to eliminate.

The same is the case with arsenic and mercury, particularly the latter. If you mercurialize a dog by friction, the animal may eliminate mercury during two months, two months and a half, even three months with complete intermission. This tardy elimination may be explained by the fact that the drug does not remain in the blood, but in the organs.—*The London Chemist and Druggist.*

